# A Dangerous Adulteration of Iodoform

**Published:** 1884-09

**Authors:** 


### A Dangerous Adulteration of Iodoform.

Dr. Biel, of St. Petersburg, calls attention to a commercial adulteration of iodoform with picric acid, which cannot be detected by the tests of Pharmacopœia. This mixture is not only poisonous, but is explosive when rubbed up in a mortar. It can be detected by the citron-yellow color it yields to a watery filtrate. If a solution of cyanide of potassium be added to the filtrate, no reaction will follow if iodoform be pure; but if there be a trace of picric acid present, the solution will, in the course of ten minutes, become brownish red (isopurpuric acid), and in a short time deposits an insoluble precipitate of isopurpurate of potassium.—*Deutsch-Amerikan. Apotheker Zeitung.*

